# What matters in health (care) universes: delusions, dilutions, and ways towards universal health justice

**DOI:** 10.1186/s12992-019-0521-7

**Published:** 2019-11-28

**Authors:** Anne-Emanuelle Birn, Laura Nervi

**Affiliations:** 1 Dalla Lana School of Public Health, University of Toronto, Toronto, Canada; 2 College of Population Health, University of New Mexico, Albuquerque, USA

**Keywords:** Universal health systems, Universal health coverage, Universal health justice, Neoliberalism, Latin America, Global health agendas, Co-optation

## Abstract

The presumed global consensus on achieving Universal Health Coverage (UHC) masks crucial issues regarding the principles and politics of what constitutes “universality” and what matters, past and present, in the struggle for health (care) justice. This article focuses on three dimensions of the problematic: 1) we unpack the rhetoric of UHC in terms of each of its three components: universal, health, and coverage; 2) paying special attention to Latin America, we revisit the neoliberal coup d’état against past and contemporary struggles for health justice, and we consider how the current neoliberal phase of capitalism has sought to arrest these struggles, co-opt their language, and narrow their vision; and 3) we re-imagine the contemporary challenges/dilemmas concerning health justice, transcending the false technocratic consensus around UHC and re-infusing the profoundly political nature of this struggle. In sum, as with the universe writ large, a range of matters matter: socio-political contexts at national and international levels, agenda-setting power, the battle over language, real policy effects, conceptual narratives, and people’s struggles for justice.

## Background

### UHC is everywhere: agendas matter

Since (re)appearing on the global health agenda in 2005 through a timid World Health Assembly resolution on sustainable health financing, universal coverage, and social health insurance [1], and then vigorously bolstered circa 2010, Universal Health Coverage (UHC) now seems to be everywhere. From the World Health Organization (WHO) to the World Bank, the Sustainable Development Goals (SDGs), the G-20, the Rockefeller Foundation and other philanthropies, as well as global, national, and regional health policy meetings and statements, and more, advocacy for UHC has reached an apparent crescendo of consensus as a priority for international development [2].

For instance, the G20 meeting in June 2019 made headlines worldwide when member nations “confirmed the importance of stronger ties to secure the necessary financial resources for UHC” [3].

A superficial reading of the voluminous production of reports, statements, and resolutions by key actors and agencies might lead policy-makers, activists, and academics alike to believe that health is (at last!) being recognized and pursued as a human right, and that the persistent calls to strengthen healthcare systems have finally become imbued in at least the discourse of these agencies (without necessarily translating into concrete changes in most international/donor aid patterns).

Today UHC is portrayed as the one and only way to improve access to health care for the half of the world’s population that lacks even minimal (not to mention comprehensive) access. Further, the 2018 Astana Declaration marking the 40th anniversary of the Alma-Ata Declaration appears to indicate that there is global agreement on a renewed commitment to primary health care as *the* strategy that will enable the goal of UHC to be reached. UHC has visibly arisen as the key idea for the present global health era, as a slogan and as a vision.

What does all of this mean for “real people,” those who are actually the targets of UHC [4]? Do we really know what UHC denotes and promotes for them?^1^ The omnipresence of the concept suggests that it is seen as the primary vehicle for contemporary global health, one that can address the shortcomings of the global disease initiatives of recent decades, which have shunted aside the fundamental role of healthcare systems. But what is UHC, where did it come from, and what does it portend? Ultimately, what matters for people’s health in our universe—or at least on our planet?

While it may seem cantankerous to question UHC (after all, how can measures to get more health care to more people be contested?), and although much ink has already been spilled on the topic, some crucial points warrant further exploration. Here we seek to unpack the drive for UHC, revisiting lively debates of recent years and paying special attention to a set of matters that merit deeper consideration in the current atmosphere of UHC verbal and policy ubiquity.

### Yet… everything is nothing: language matters

The word *universality* is expansively defined as “pertaining to the whole of something specified; occurring everywhere” [5]. In the area of mechanics, universality means “allowing *free movement* in any direction” (as per the 1670s term “universal joint”). This connotation implies that what is universal need not be impeded by constraints, be they ethical principles, values of accountability, or other factors.

Moreover, the term *coverage* is problematic in its very origins. Etymologically traceable to a “charge for a booth at a fair” [5], its more recent use stems from the early twentieth-century US insurance industry, referring to the “amount of protection given by a policy.” However, coverage of what, for whom, how, and so on has remained vague.

Clearly, parsing of the expression UHC is both illuminating and troubling. Since its resurgence, observers have been citing the ambiguous meaning(s) of the term UHC and its component parts: despite its “apparent momentum… the goals of UHC’s proponents are unknown except in broad senses” [6]. Especially in the United States, UHC appeals to the idea of health insurance for everyone. UHC is also viewed by some as a principle/guarantee of healthcare services for all (gratis or at low cost), or to welfare-state-like national health systems and services.

Such equivocation has prompted “an impassioned debate among today’s global health community about how UHC should be operationalized in low- and middle-income countries,” particularly in Latin America [7]. Critical analysts have underscored how these ambiguities have both led to diverse governmental and civil society interpretations and policy approaches *and* enabled co-optation by powerful forces of the long-time progressive agenda for public sector-based comprehensive, equitable, and accessible healthcare systems [8–11].

Beyond the definitional predicaments surrounding “universal” and “coverage,” there is a (not so) surprising silence: among mainstream UHC champions, the matter of what comprises/constitutes health is largely sidestepped. Does “health” solely signify “health care”—or does health writ large figure into this issue, as per contentious discussions around the “right to health” versus the “right to health care”? Amid such questions, a wide-ranging debate is sorely needed.

Further, although the most critical analysts have advocated for broadly addressing (not just rhetorically invoking) the political, commercial, and other societal determinants of health [12, 13] instead of the exclusive emphasis on UHC [14], the terms of the debate remain framed by a reductionist healthcare-centered vision. This stance (i.e. that health derives from health care), advanced by the dominant players around UHC (WHO, the World Bank, the Rockefeller Foundation, etc.), means that almost all of the focus has been around the *language* of universal and coverage, with little attention paid to health per se. This mainstream take on UHC turns societal and social concerns into narrowly medical ones and subjects them to individualized, technical, and de-politicized interventions.

In taking deliberations around UHC to the next, stratospheric, level, we propose, and will circle back to, the need to revive a broad understanding of health (not solely as healthcare access, even as this remains a critical component) while recognizing the deeply political and politicized nature of the struggles inherent to advancing *universal health justice* as the core of the global health agenda.

## Main text

### How did we get here? An emerging and re-emerging concern: historical context matters

The call for UHC is not new. To be sure, while the particular configuration of this term is recent, the concept and pursuit of UHC date back to at least the nineteenth century, traceable to militant workers’ movements and fiery policy debates. This activism spanned workers’ mutual aid societies (cooperative, non-profit insurance arrangements for particular ethnic groups, occupational clusters, and geographic localities) across Europe and Southern Cone countries, as well as bottom–up and top–down governmental efforts (social movement-based and more cynical repressive gestures) aimed at extending the reach of—and rights to—publicly-funded (and -delivered) healthcare services, first for urban industrial workers and civil servants, and then for greater and greater swathes of the population, eventually including family members of these groups, rural agricultural workers, the elderly, informal sector workers, and others [15].

The resulting arrangement of healthcare services, and rights/access to them, depended both on timing and context. For example, in the 1880s, the universal, non-profit private health insurance arrangement in Bismarck’s Germany offered a carrot to laborites, while reining in their activism [16]. Great Britain saw multiple efforts, building upon mutual aid societies starting in 1911 but only gradually expanding upon who and what was eligible until the sacrifices and sense of unity during World War II enabled a post-war Labour government to push through a public national health service (although—unlike Canada’s subsequent, if now battered, attempts—never fully abolishing a private sector). In the 1920s, the Soviet Union’s post-revolutionary, centrally administered socialist health system integrated healthcare services, research, pharmaceutical production, and public health, and came to serve as a model for socialist states.

As early as the 1920s most Latin American countries, including then-industrializing Argentina, Chile, and Mexico—each undergoing a flurry of state-building efforts—witnessed the beginnings of healthcare systems within social security expansion. However, almost all of these systems remain/ed. highly segmented, with distinct schemes and services for different industrial sectors and population groups (including informal and agricultural workers), plus persistent, all but untouchable, private healthcare arrangements for elites.^2^ Both Chile and Costa Rica sought to rationalize this segmentation in the mid-twentieth century; and after its 1960s–1980s dictatorship, Brazil responded to a wide social mobilization, creating a unified public healthcare system (albeit retaining a powerful private sector). Only post-revolutionary Cuba achieved a socialist healthcare system capable of resolving the inevitable inequities engendered by separate, or what are sometimes referred to euphemistically as “pluralistic,” systems [20, 21].

Depression-era New Zealand and Sweden managed to harness worker solidarity into among the most far-reaching national health services in the context of burgeoning welfare states. Sidelined from these developments were many former colonies across Africa, the Caribbean, and Asia, virtually all of them financially strapped given the terms and cost of decolonization, with only a few (like Barbados and Sri Lanka) managing to channel liberation struggles into public population-wide healthcare systems. Elsewhere, civil servants and (some) industrial workers were among the few groups to enjoy healthcare entitlements. Similarly, in the USA’s private insurance-driven (though paradoxically majority publicly-financed) healthcare market, only seniors, persons with long-term disabilities, and veterans attained (mostly) publicly-funded health coverage. Additionally, certain low-income groups and Native Americans have come under nominally publicly-funded and -delivered arrangements, but with much lower access and quality. Throughout these different eras, the International Labour Organization (ILO) and other multilateral agencies have advocated for countries to extend social security insurance [22].

Yet even in those countries that putatively achieved “universal” public-sector healthcare rights and access, especially in the post World War II era, *universality (has) remained an aspiration rather than a reality***.** Whether particular types of care are not, or are inadequately, covered—for example, LGBTQ health needs, therapies for rare diseases, public health, expensive care (e.g. for treating cancer), reproductive health services (birth control and abortion)—or some populations are left out, such as temporary/new residents, refugees, and migrant workers, or Indigenous populations, whose healing traditions have long been marginalized and excluded, no system or country should have the hubris to declare that it has achieved “universality.” In many settings, segmenting the population is a legacy of sector-by-sector labor struggles—or an intentional effort to divide populations, as in the USA, with means-tested, decentralized, limited, and underfunded services for the indigent under Medicaid, as opposed to Medicare’s better-funded (although premiums, deductibles, coinsurance, and exclusions can still result in exorbitant out-of-pocket expenses), more comprehensive coverage for seniors and people with long-term disabilities.

Across Europe, the Americas, and parts of Asia in the late 19th and into the 20th centuries—and for Africa, the Caribbean, South Asia/some of East Asia in the decolonizing post-Cold War era—these precursors to contemporary UHC efforts marked an arc of struggle for social rights, public healthcare systems, and welfare states. Cold War tensions between capitalist and communist blocs sometimes spilled over into debates around social security vs. socialism, often serving to advance healthcare rights. Notwithstanding the shortcomings of these efforts, many did, as per Martin Luther King’s dictum, begin to bend towards equity in the context of public, comprehensive, population-wide healthcare systems. Especially in Scandinavia and in socialist countries, public and seemingly universal benefits grew expansively, consistent with the general trend of redistributive, socialist, and social democratic states having better population health indicators than other societies [23]. Nonetheless even in Sweden, for example—whose universal healthcare system has long been considered one of the most egalitarian in the world—sexist, racist, and classist health policies and institutions remain. This is witnessed by delays in care and second-class care reported by immigrants [24] and by market-oriented primary healthcare reforms that privilege access to care for the affluent and healthy [25]. All told, universality, even if invoked as such, has never been reached, because of who and what are left out of policy design and policy outputs/outcomes. Universality remains an elusive if admirable goal.

Even so, and despite the exclusions and stratification characteristic of much of the Third World and parts of the First (especially the USA), efforts at creating publicly-financed and operated healthcare systems made undeniable gains until the 1970s. Marking the apex of these struggles at the international level, and their turning point (with dashed hopes), was the International Conference on Primary Health Care held in Alma-Ata, Kazakhstan (former USSR), in 1978 and the movement surrounding it [26]. The effort to move from disease control to the broader right to health, from top–down to community-based approaches to health—all in the context of a New International Economic Order, upending existing power asymmetries between First World and Third, between capital and labor, etc.—might have become revolutionary indeed.

But the decreasing profitability of capital against the mounting gains of crisscrossing people’s movements—for the rights of women, Indigenous groups, LGBTQ, the environment, workers—amid ever-more inclusive and robust welfare states in high-income countries (HICs), as well as in a growing number of low- and middle-income countries (LMICs), led capitalist elites and their political allies to engage in a frontal attack. Starting in the late 1970s and heightening in the 1980s, enabled by the waning threat of the Soviet model, the world order was re-oriented toward business interests—a process of neoliberal globalization aimed at making capital profitable again. Initially led by neoconservative governments in the UK and USA, this transformation to a neoliberal phase of capitalism [27] (neoliberalism for short) has involved infusing “free” market ideology throughout the world through a series of steps, from currency liberalization and debt crises, to rapacious forced loans and debt servicing (bailing out the HIC private banking sector), and financialization (the process through which the financial sector increases in size and influence in relation to the overall economy)—each generating untold social misery, including massive effects on health and the marketization of health care.

As succinctly put by Wendy Brown:

Neoliberalism—the ideas, the institutions, the policies, the political rationality—has, along with its spawn, financialization, likely shaped recent world history as profoundly as any other nameable phenomenon in the same period, even if scholars continue to debate precisely what both are. … Neoliberalism is most commonly associated with a bundle of policies privatizing ownership and services, radically reducing the social state, leashing labor, deregulating capital, and producing a tax-and-tariff-friendly climate to direct foreign investors [28].

There is a substantial body of evidence that connects deteriorating health outcomes to neoliberalism, due to the associated rise in economic inequality, insecurity, and poverty [29] and mediated by the worsening living and working conditions produced by neoliberal policies, including the effects of labor precarity and austerity policies on physical, mental, and behavioral health. Further, as we shall see, UHC—although portrayed as an antidote to the consequences of neoliberalism for healthcare access—is itself shaped by, and a product of, neoliberal ideology and policies.

Painfully illustrating the real effects of this onslaught in LMICs, Latin America became a laboratory for neoliberalism. This started with Augusto Pinochet’s US-backed 1973 military coup and decades-long dictatorship in Chile, which piloted economic liberalization and privatization of public services. By the 1980s, a regionwide (soon to be LMIC-wide) debt crisis generated a “lost decade.” Interest-rate hikes by major banks led to a doubling of Latin America’s debt burden in just three years. Beginning with Mexico in 1982, dozens of countries within and beyond Latin America defaulted on private loans in rapid succession [30, 31]. The International Monetary Fund and World Bank orchestrated structural adjustment loans to “help” debtor countries reduce deficits and meet debt-servicing burdens by forcing a series of neoliberal reforms. These obligatory economic reforms (quid pro quo loan “conditionalities”) were designed to fuel low-cost exports and open domestic economies to foreign direct investment (FDI). Measures comprised: drastic cuts to social spending (including to the already-underfunded health sector); removal of agricultural subsidies; ratcheting back of labor protections; deregulation of mining and other industries; lifting of restrictions on foreign investment and banking; trade liberalization; currency devaluation; privatization of government services and state-owned assets; and imposition of user fees for school attendance and health services [15].

These much-reviled reforms damaged health, not only through corroded quality and huge declines in access to care, but also through deterioration in education, wages, neighborhood conditions, worker safety, and a host of other societal protections. Exacerbating pre-existing inequities and problems of fragmentation, neoliberal reforms—under the advisement of international financial institutions (IFIs)—sparked a wave of social-security and health-system reforms in Latin America and across the Third World [32].

The specific and often enduring effects on the healthcare sector have been wide-ranging, as illustrated by the Latin American experience. On one level, increased entry of financial capital stimulated greater private health insurance coverage for the healthy and wealthy (entrenching the long-standing practice of excluding “high-risk” individuals); by the 1990s there was soaring FDI in social security systems (feeding on health insurance and pension “markets”) serving middle class and formal sector workers. Simultaneously, Big Pharma increasingly displaced domestic generic and public drug manufacturers and distributors. On another level, state health systems became privatized “from within”—with public funds used to contract private hospitals and providers, outsourcing of management and human resources, and subcontracting to private entities of profitable services, including laboratories and pharmacies, as well as food services, cleaning, and patient transportation. On yet another level, policy-makers turned to user fees in order to increase revenue and reduce demand for state-funded health services and institutions. While “The proponents of these measures touted their efficiency and transparency, … they have produced opposite effects: waste, unnecessary expenses, growing inequities and corruption” [10].

Such ruthless policies spurred persistent and widespread resistance worldwide, perhaps nowhere more than in Latin America, by the early 2000s reaching ballot boxes across the region in an upsurge for change. The much-touted Pink Tide of socialist-leaning or social democratic movements, political parties, and elected governments used sizeable commodity-boom earnings to invest in a host of policies aimed at improving living and working conditions (thereby improving health)—from living wages to the enforcement of labor laws, and including greater access (and in some cases rights) to healthcare services. Concerted progressive policies ranged from anti-poverty programs such as *non*conditional cash transfers^3^ (Uruguay), to integrated nutrition, primary health care, and cash-transfer programs (Brazil), progressive tax reforms and a unified public health insurance financing pool (Uruguay), neighborhood-based primary-care clinics (Venezuela), intercultural healthcare services (Bolivia), and increased access to an integrated network of health services and effective intersectoral actions for health (El Salvador) [33].

In some cases, progressive health reforms predated the arrival of left-leaning governments to power, as with Brazil’s investing in a unified public healthcare system. In others, neoliberalism was embodied in contrary health reforms through UHC-style “structured pluralism” approaches (Colombia, Mexico, Peru), continuing (albeit expanding) highly inequitable segmented and stratified healthcare arrangements [34, 35].

Although measurably bettering health and social conditions, Pink Tide efforts did not fundamentally upend the capitalist basis of these societies because most of the social investments came not from redistribution or tax-code reforms, but from commodity earnings (even in Venezuela, Barrio Adentro was never integrated with the public system, and the capitalist economy has remained in place). Moreover, persistent asymmetries of power enabled a shockingly rapid retrogression of these reforms in the wake of the commodity bust, with a return to conservative rule in recent years (which, as this article was going to press, was being contested by the election of anti-neoliberal governments in Mexico and Argentina and by massive popular demonstrations and mobilizations against neoliberal policies in Ecuador, Chile, and elsewhere).

Even before the Pink Tide, the suffering under neoliberal globalization had not gone unnoticed by IFIs; however, their remedies typically reinforced the very policies they purported to address. The World Bank sought to give a “human face” to its loan remedies, creating an alphabet soup of partial debt forgiveness programs—without de-attaching conditionalities, leaving countries tethered to the same economic precepts that were strangling them. The WHO’s 2000–2002 Commission on Macro-Economics and Health underscored the issue of ill health in instrumental terms—as draining productivity and causing impoverishment—but failed to grasp the role of poverty in generating ill health in the first place [36].

Emblematic of the neoliberal response by international agencies, UHC in many ways arose out of the preoccupations raised by this Commission. Meanwhile, the 2003–2008 WHO Commission on Social Determinants of Health (SDOH) sought to recapture the agenda by focusing on social injustice, unequal distribution of power and resources, and the need to improve the day-to-day living conditions of “the other half” of the world’s population who had seen their circumstances devastated under neoliberal ascendance. But the push for SDOH remained at a largely technocratic level and was soon overshadowed by philanthrocapitalist encouraged top–down (vertical) disease initiatives [37].

Fast forward another decade and these mixed aims—a nod to rights amid the deterioration of social conditions, but with assurance of financialization—are now fully inscribed in the SDGs of the 2030 Agenda for Sustainable Development, adopted at a UN Summit in September 2015. Goal 3, Target 3.8 puts UHC among the global priorities for development: “Achieve universal health coverage, including financial risk protection, access to quality essential health care services and access to safe, effective, quality and affordable essential medicines and vaccines for all” [38].

In this short definition, and in the monitoring indicators defined a year later, the two fundamental elements of UHC are highlighted: access to essential health services (“essential”: a limited rather than comprehensive package of provisions); and financial risk protection, understood as the proportion of the total income of each household spent on health care—aimed at preventing /limiting medical bankruptcy/impoverishment, while ensuring new markets for insurance capital.

In sum, UHC’s genealogy—despite policy-makers’ efforts to invoke public, rights-based healthcare systems as forerunners—reveals a massive dilution of the progressive health agenda: UHC stems from, and is consistent with, the neoliberal turn in global capitalism.

### Unveiling the co-optation and perils of UHC beyond discourses: policy matters

The war of words is important: as noted by Foucault and many others, power is evidenced and manifests in discourse, which in turn profoundly shapes (and is shaped by) how people’s preoccupations are defined, political priorities are set, and policy-makers establish guiding agendas.

And yet, deliberation is not enough. When the debates (and debaters) are exhausted and even once a discourse of (healthcare) rights and (healthcare) justice takes hold, as it seems to have, the realities of governing and policy become the crucial space for attention—and struggles (see below).

Delving into how and why UHC is not equivalent to public and universal healthcare systems necessitates far more than rhetorical unpacking. Instead, as made clear in the scholarly literature, it requires an unveiling of the concrete policy agendas, implementation consequences, and further implications embedded in this trend. Here we review a selection of critical analyses showing why UHC is a problematic approach and what assumptions undergird the policies and practices it spawns.

Building on the ambiguities cited above, Lethbridge emphasizes that WHO and the World Bank Group’s promotion of universal health *coverag*e—as opposed to *provision*—“involves the creation of health insurance schemes which allow people to access health care facilities run by public, private and not-for-profit sectors” [39] and adds that universal healthcare provision (or universal health systems [UHS]: publicly financed and delivered, single-payer healthcare systems), on the other hand, “dictates that the government guarantees the actual provision of health care services to everyone, irrespective of income, status, etc., rather than offering what is in effect hypothetical ‘access.’” The “hypothetical” access offered by the UHC model is another way of saying that coverage only ensures nominal, not necessarily effective or realizable, access to health care.

Moreover, despite the assertion that public sector provision is included in the UHC mix, what it heralds in terms of actual policy is the extension of insurance to those not currently covered through partial financial protection via (circumscribed) packages of “essential” services, often purveyed in the for-profit sector. This harks back to the 1993 World Bank’s World Development Report’s “Investing in Health” neoliberal prescription for scaled-back public services, opening the door to private investment, and advocacy for out-of-pocket payment (user fees) for healthcare services.

Amplifying the critical analysis, Giovanella and colleagues [40] compare and contrast UHC-based and UHS-based models, identifying a range of divergent features. What follows is a non-exhaustive summary of what these authors argue based on their comprehensive review of the literature^4^:

- The UHC approach conceives of health as a commodity; UHS recognize health as a human right.
- In UHC, the role of the state is minimized, restricted to the regulation of the healthcare system, with explicit separation of financing/purchasing and service functions. UHS, by contrast, are based on principles of social welfare, wherein the state is responsible for the funding, management, and delivery of health services.
- As to funding, UHC is based on the pooling of public and private funds (insurance premiums, social contributions, philanthropy, taxes); UHS are based on public funding via tax revenues (general taxes and social insurance contributions).
- Regarding the underlying rationale for each reform, UHC subsidizes/incentivizes demand for health insurance purchase via delimited packages of services and targeting of (some of) the poorest; UHS subsidize supply to guarantee equitable access to the entire population.
- Concerning eligibility/entitlement, UHC systems create segmented access based on enrollment within particular insurance schemes (private or public); UHS pursue universal access as a condition of citizenship or residency.
- In terms of efficiency, the UHC approach raises operational and administrative costs, leading to higher total expenditures on health; UHS maintain lower administrative and operational costs, reduced unit costs due to economies of scale, and lower (or at least more equitably distributed) total expenses due to greater regulation of supply.
- UHC arrangements are fragmented, providing only selective packages of primary healthcare services; UHS are organized through networks of territorially-based, comprehensive primary health care.
- UHC is focused on individual care and biomedical services and is separated from collective care; UHS seek to integrate individual care and public health actions, as well as integrating health promotion, prevention, and curative care.
- Lastly, UHC marginalizes the SDOH approach; UHS incorporate societal determinants of health and call for intersectoral action.

These differences are not simply abstract policy matters but translate into real pocketbook and access issues. Insurance arrangements that involve premiums, co-insurance and co-payments, and often sky-high deductibles may ostensibly increase UHC, while impeding actual access—both because the costs themselves may be prohibitive, with household health resources spent on payments rather than services, and because the modus operandi of UHC-based systems is to minimize health-services use through barriers to care. Paradoxically, as we will see, opposing pressures for increased use of certain products and services are also a factor.

In the end, as seen in Latin America and beyond, the results of the expansion of UHC-based health arrangements (as opposed to UHS) have been grim, negating the touted gains of these reforms. Far from being a major accomplishment for global health and equity, UHC represents a continuation (wrapped in emperor’s clothes) of (more) business as (than) usual since the rise of the neoliberal phase of capitalism and the assault on the long struggles for health and healthcare justice.

To contend otherwise would be a delusion.

In a prior analysis, we argued that UHC represents one of the most cynical and effective contemporary instances of co-optation of the global health equity agenda [10]. Despite having recommitted itself to the Alma-Ata principles in 2008, WHO fully endorsed UHC two years later, amid the Great Recession and in a context of rising healthcare costs and continued LMIC healthcare system disarray “following decades of neglect and downsizing.” We cited well-grounded studies showing that “through UHC, insurance corporations gain access to public revenue streams (social security contributions and taxes) that finance contracts to provide a set of services to the previously uninsured,” and noted with dismay that those newly covered under UHC schemes are “overwhelmingly economically precarious” yet are often legally required to spend large proportions of their earnings to pay a range of user fees and new taxes regardless of their precarity, and that those even more vulnerable—informal sector workers—may be excluded from “universality” altogether [10].

Acknowledging the menaces of UHC policies, some agencies have sought to preserve the message of comprehensive, equitable approaches. The Pan American Health Organization’s (PAHO’s) “Strategy for Universal Access to Health and Universal Health Coverage,” adopted in October 2014, recognizes that these principles “imply that all people and communities have access, without any kind of discrimination, to comprehensive, appropriate, and timely, quality health services determined at the national level…as well as access to safe, affordable, effective, quality medicines…” [43]. Taking up the term “universal health” to overcome the dilemmas over *coverage*, PAHO endorses a “multisector approach to address the social determinants of health and promote a society-wide commitment to further health and well-being” [43].

Nevertheless, in refraining from discussing the roots of social and health injustices, or the power relations that impede the fundamental changes needed to address these injustices, PAHO enshrines a functionalist UHC approach. Incorporating a discourse of health justice without actions to remedy *in*justice is insufficient. Inequities are not genetic: they are produced by societies in which a small and powerful elite garners massive advantages, excluding most of the population.

While functionalist approaches can enable the *identification* of inequities, they draw attention to the existence of inequities without actually addressing them. Ultimately, such approaches attribute inequities to unavoidable societal malfunctions, not to a system designed to perpetuate asymmetries of power and maintain structures that benefit elites to the detriment of the majority. As such, even PAHO’s aspirational language is unable to circumvent the deep flaws of UHC.

To reiterate, a critical mass of research findings has indicated the inadequacies of UHC in terms of equity, fairness, social justice, and health itself [39, 40]. It is essential to consider UHC *not* as an innovation that has magically appeared to address/resolve the woes of inaccessible and inequitable health care (not to mention health) of unspecified genesis, but as the logical outgrowth of four decades of neoliberal capitalist (health) ideology and associated policy-making. Notwithstanding the clever semantic flourish of incorporating the word “universal,” UHC effectively reproduces the features of neoliberalism that have plagued healthcare systems, health, and overall societal well-being over recent decades: proliferating user fees, privatization and outsourcing, public subsidies to the for-profit sector, subcontracting of public roles and jobs to private interests, greater precarity and loss of union protection for health workers, increased market entry for profiteering corporate interests (e.g., insurance companies and pharmaceuticals), carte blanche to FDI with a bare minimum of regulation/oversight—and more.

In sum, UHC represents not a break with the past, but rather the fruition of a long assault on public healthcare systems and welfare states, and, therefore, on health. It is only the de-contextualized and de-historicized portrayal of UHC that makes it seem revolutionary instead of just one more brick in the mansion of neoliberalism. Unfortunately, many health advocates and activists, pleased at what they envision as a step towards universal and public healthcare systems, have not fully recognized the nefarious underbelly of UHC.

Transcending the debates and dilemmas around UHC versus UHS is an additional element little discussed in health-policy circles. It is critical to take into account that, in conjunction with the rise of neoliberal globalization in the 1980s, US (later global) biomedicine began to be “transformed from the inside out through old and new social arrangements that implement biomedical, computer, and information sciences and technologies to intervene in health, illness, healing, the organization of medical care, and how we think about and live ‘life itself’” [44].

Biomedicalization generates an internalized “self-control and [health] surveillance” regime, whereby healthy individuals are encouraged to undergo health “enhancements” through unnecessary and often dangerous procedures and products, such as vitamin supplements and cosmetic surgery. As Iriart and Mehry argue, biomedicalization involves not only “defining, detecting and treating” illness processes, but also “being informed and alert to potential risks and conditions that could lead to disease” [45]. This frequently takes the form of disease-mongering—direct or indirect marketing or preying on subject populations, be they youth and young parents on social media, or older groups affected by more traditional channels. Examples of how this process affects real people include the assignment of “at-risk” or “sick” labels to otherwise healthy individuals when the threshold values of what is deemed to constitute hypertension, high cholesterol, hyperactivity, anxiety, depression, or overweight/obesity are arbitrarily lowered, prompting the ordering/prescription/consumption of tests and drugs “of dubious efficacy and in many cases iatrogenic” [45].

These processes are driven by corporate profiteering, and abetted by WHO and other international agencies propagating a noncommunicable disease crisis [46]: disease-mongering and global agenda-setting combine with internalized subjectivity to “pre-disease,” creating huge marketing possibilities. As a result, healthcare systems around the world are beleaguered by soaring costs due to over-diagnosis, over-prescribing, and over-treatment, with severe consequences for the well-being of populations and the financial sustainability of healthcare systems. Meanwhile, the production and distribution of ultra-processed food and beverages directly related to the worsening of key health indicators remain largely unregulated.

Ironically, but not unexpectedly, biomedicalization has also provoked deep suspicions, often wrongheaded and uninformed, about the safety and purview of the mainstream biomedical establishment, resulting in the burgeoning appeal of “alternative” medicine in both HICs and LMICs where traditional healing cultures have long been disappearing.

Moreover, the realm of biomedicalization reveals a profound and under-discussed paradox relating to UHC. If, as per the above, it is in the interest of the private insurance sector to minimize people’s access to healthcare services in order to maximize profits, the reverse is true for industrial (medical) capital (Big Pharma, Big Diagnostics, Big Devices, etc.): increasing access to services and products is both necessary and desirable, regardless of the funding stream [45]. Such paradoxes between the different segments of capital, even as at times they also join forces, make the analytic task and points of resistance extremely complex.

Although debates around UHC have centered on healthcare services and systems, the larger environment of biomedical hegemony and of capitalist extraction exists both inside and outside the boundaries of what are typically understood as healthcare systems. Thus, it behooves us to recognize the struggles ahead as occurring both within the health sector and well beyond it.

## Conclusions

### Deconstructing is not enough: politics, power relations, and struggles matter

If “being insured does not mean guaranteed access to health services” (let alone a comprehensive array of services) and if “the UHC model increases segmentation and crystallizes social stratification and inequities in access to health and health conditions” [40], how can the large majority of activists, communities, health workers, and scholars committed to health equity and social justice escape the dilutions and delusions of UHC? How can the terms of the debate be recast to—together with the necessary struggles and actions—put us all on the road towards universal health justice?

Our invocation of the term “universal health justice” rests on the conviction that each and every individual/community/population, regardless of who they are or where they come from, deserves equal rights and equitable outcomes, including the right to health (and health care), and the power to exercise those rights. Drawing on the work of countless social justice-oriented activists, policy-makers, academics, investigative journalists, and others [14], we hold that the path towards universal health justice requires: an understanding of how the structural determinants and determination of health [47] operate within each context and globally; identification of what policies and social forces reinforce the associated asymmetries of power; organizing to remove structural impediments to health (explicitly including racism, sexism, xenophobia, poverty, hetero-normativism, classism, denialism of climate change, among others); defining specific local and global strategies to create equitable and fair living and working conditions within and across societies; and building equitable and participatory healthcare systems for all.

#### Beyond UHC mania

Of central importance is not getting lost in the technocratic battles created by those who frame the debate [48, 49]. We join the chorus of actors from LMICs/Global South and allies who call for the political to be extracted from the technocratic. Even an aspirational understanding of UHC is far more than a matter of extending the right “model.” This is a fundamentally political issue about how resources are harnessed and distributed and affect people’s lives and does not just involve the balance sheets of decision-makers or international bureaucrats. Here, the questions concern the political spaces afforded governments seeking to be accountable to people’s needs—be it governments heavily reliant on foreign donors, or those with putatively more “sovereignty” but having virtually no possibilities of implementing the progressive platforms on which they are elected because of the constraints (financial, political, and more) of the neoliberal capitalist global order. For these peoples and societies, it is politics that matters, at the local and global levels, far more than the war of words.

#### Adiós a la nostalgia

Above all, it is crucial that progressive-minded critics of UHC not wield a superficial nostalgia for welfare states of yore—we should not retreat to a defensive stance or wear blinders to the multifarious and deep limitations of even the most expansive welfare states of the past. This is undoubtedly a complicated task, given how many rights, services, and protections have been clawed back in recent decades in both LMICs and HICs. Building anew will require aspirations and values that strive to overcome the constraints and limits of what have often been welfare states designed largely by and for (mostly) men, civil servants, settler-colonialist groups, those of dominant ethnicity, and the industrial working class [50].

UHS, as outlined above (comprehensive healthcare access as a right, with unified, integrated, and publicly financed and delivered healthcare services) are worth fighting for—but they will have to be envisioned and structured anew, to break with embedded forms of oppression and inequities from the past.

Among the knowable requisites for paving the long road to UHS oriented to universal health justice are: assurance of political and financial viability (with global cooperation, if necessary); bona fide representation and participation of the people and of healthcare workers^5^ in decisions and management; constitutional and tax-code reforms, and reforms to prevent illicit financial flows that drain domestic coffers; addressing the geographic, social, and system-level barriers to health care (inequitable quantity, distribution, and networks of health services, institutional and societal racism, xenophobia, sexism, aporophobia, queerphobia, inadequate or unaffordable transportation, etc.); multisectoral planning and coordination; ensuring, as a matter of human rights, women’s full access to reproductive health services, including abortion; critical evaluation, monitoring, and forestalling of the negative effects of corporatization and biomedicalization on population health and health systems (and inclusion of non-biomedically-based health models, within a perspective of interculturality); global (health) agencies and policies that respond to articulated and contextualized needs and priorities from below, instead of health-policy agendas crafted on the interests of corporations and their political partners; and addressing gender and social inequities within the healthcare workforce.

#### Continue building counter-hegemonic epistemological and policy paradigms

We recognize the ongoing role of evolving worldviews and policy frames that articulate health as the product of the conditions of life, work, the environmental viability of the planet, and the intersectionality of class, gender, and race/ethnicity. Part and parcel of this effort is understanding the power dynamics that underscore the ways in which economic and political resources and power are themselves appropriated by and concentrated among elites and dominant structures, including via a hegemonic biomedical model. Nowhere is this more evident than in the contemporary resurgence of authoritarian governments as a “therapeutic” response to widespread deterioration in social conditions, employment prospects and security, social protections and rights, health and life expectancy, and morale—whether in the USA, Brazil, Hungary, or India. Gramsci reminds us that day-to-day struggles reside not only at the level of material circumstances, but also in the ideological battlefields around what constitutes a desirable, ethical, and fair society amid a “contradictory consciousness” that pervades thought processes and influences people, often against their own objective interests. It is in this context that the combined interests of capital and right-wing hate-mongers in many societies have revived the attractiveness of fascist parties and beliefs, often goaded on by conservative evangelical religious forces, in which members of the working class (mostly men) rally their resentment against all possible “others”—especially, but not exclusively, immigrants, women, people of “non-dominant” race/ethnicity, LGBTQ, Indigenous groups, and low-income populations—while never addressing the true perpetrators of declining quality/conditions of life: ever-greedy capitalist interests [52].

Translated to the global health arena, counter-hegemonic paradigms must include analysis of the embedded assumptions in the many global policies that may be characterized as “Grand Plans from Above” (UHC, as well as GH2035, etc.), in which (indisputably growing and persistent) health inequities between countries, social classes, and groups are cited—but then normalized, naturalized, depoliticized, and ultimately discarded. Not only do these Grand Plans from Above fail to recognize or address the roots of these inequities, they also blame the public for their own “poor choices,” viewing social and health injustices (particularly in LMICs) as inherent to their own contextual realities. Oft mentioned are the role of corruption (which also affects the USA and other HICs) and the influence of elite/corporate interests on policy-making (legalized in the USA through political lobbying), with no acknowledgement of the role of colonialism/imperialism in the making of abysmal inequities in the first place, and in the continued extraction of profits and resources, or of the concentration of power in the hands of global and local elites.

#### In whose universe? Towards transforming the global political order: real people, real actions, and real movements matter

We have raised a few points, admittedly incomplete, aimed at taking back the (terms of the) debate over what we call “universal health justice.” However, we recognize that the real political challenge lies in achieving the transformation of the global political and economic order.

In examining and engaging in the “hows” of achieving this transformation from our political vantage point in the global health arena, it is important to bear in mind that struggles for health justice, social justice, and economic justice go together—that struggles for just (fair) health policies at the level of healthcare systems are part of larger struggles for equitable social and economic policies. Striving for socially just economic/financial and social policies writ large is central to any effort towards universal health justice.

If anything has been learned from the neoliberal period of global capitalism of the past four decades in Latin America and the conservative resurrection in the last few years (following the Pink Tide), it is that “even the most profound health and social justice achievements may be reversible” [10]. This is a poignant reminder that historic gains are and will remain provisional as long as the construction of democratic societies continues to rest solely on the idea of a clean electoral system—without addressing and fundamentally transforming the role of real people in the construction of power within and across societies on an ongoing basis. Social participation must not be a checklist or an afterthought that tinkers at the margins while continuing to reproduce power asymmetries: without true social accountability of government actions and international (financial and governance) rules and arrangements, in all their dimensions, there can be little real or lasting change.

The necessary transformations will require sustained, indefatigable, and relentless commitment and mobilization that continuously recognizes and addresses the injustices highlighted above, drawing on a deep conceptual understanding, and enabling the development of real, feasible, if ambitious, actions to confront these injustices. It is crucial to learn from and practice solidarity with the many inspiring and persevering movements, especially in the Global South, that struggle for justice—including decent-work movements, youth movements, women’s movements, Indigenous rights movements, anti-racism movements, anti-imperial and anti-militarism movements, environmental justice movements, tax and banking/financial system justice movements, trade justice efforts, movements to combat resource extraction and climate change, and so many others. The struggle for universal health justice should not be separate from these movements: a universal vision for health justice demands universal actions.

## Data Availability

Not applicable.

## Abbreviations

FDI: Foreign Direct Investment
HICs: High-Income Countries
IFIs: International Financial Institutions
ILO: International Labour Organization
LGBTQ: Lesbian, Gay, Bisexual, Transgender, and Queer
LMICs: Low-and Middle-Income Countries
PAHO: Pan American Health Organization
SDGs: Sustainable Development Goals
SDOH: Social Determinants of Health
UHC: Universal Health Coverage
UHJ: Universal Health Justice
UHS: Universal Health Systems
UK: United Kingdom
UN: United Nations
USA: United States of America
USSR: Union of Soviet Socialist Republics
WHO: World Health Organization

## Footnotes

1 Here we use “them” in an “othering” sense, as UHC policies and schemes are rarely relevant or applicable to decision-makers, academics, and international agency experts.

2 The extreme segmentation of healthcare systems in Latin America is not a question of policy design: it is the product of structural economic heterogeneity. Within most countries in the region, there are vast differences in productivity levels and labor-market features by economic sector, resulting in enormous inequalities in remuneration, benefits, and, ultimately, relations among state, market, and population [17]. Although inequality has at times decreased (as during the first decade of this millennium), Latin America—with its historical and structural inequalities—remains the most unequal region of the world, followed by sub-Saharan Africa [18, 19].

3 It is essential to distinguish between nonconditional transfers going to all those who meet poverty criteria, and conditional cash transfers based on a mandatory and paternalistic quid pro quo, such as proof of school and health clinic attendance, regardless of the quality and accessibility of these services.

4 There is a vast literature analyzing the equity, efficiency, fairness, and health outcomes of for-profit versus non-profit healthcare delivery in the United States, including these two classic reviews [41, 42].

5 A crucial, if oft-ignored, dimension of UHC and even many UHS concerns the role of healthcare workers, who play a central role in any healthcare system, but are increasingly subject to authoritarian management, ever-deteriorating labor relations and work conditions, and precarious employment, particularly in LMICs. Despite this ongoing dehumanization of health workers, “attempts to incorporate social participation into health systems almost always bypass health workers” [51].

## References

[1] WHO. Resolutions of the 58^th^ Session, World Health Assembly, 16–25 May 2005. https://www.who.int/mediacentre/news/releases/2005/pr_wha06/en/

[2] Bloom G, Katsuma Y, Rao KD, Makimoto S, Yin JD, Leung GM (2019). Next steps towards universal health coverage call for global leadership. BMJ..

[3] NHK World Japan. https://www3.nhk.or.jp/nhkworld/en/news/20190629_05/. Accessed 28 June 2019.

[4] Philips M, Voute C, Akerfeldt K. An agenda to tackle the politics of real problems and real people. In: Kirton J, Kickbusch I, editors. Health: A Political Choice: Delivering Universal Health Coverage 2030. Global Governance Project: GT Media Group, London; 2019. p. 100–1.

[5] Online Etymology Dictionary. https://www.etymonline.com/word/universal. Accessed 6 June 2019.

[6] Bump JB (2017). The long road to universal health coverage: a century of lessons for development strategy.

[7] Gorsky M, Sirrs C (2018). The rise and fall of “universal health coverage” as a goal of international health politics, 1925–1952. Am J Public Health.

[8] Chiriboga D. What is behind the sudden global marketplace call for universal health care: co-opting in the making. PEAH– Policies for Equitable Access to Health. 2014. http://www.peah.it/2014/04/what-is-behind-the-sudden-global-marketplace-call-for-universal-healthcare-co-opting-in-the-making/. Accessed 15 Sept 2015.

[9] Waitzkin H. Universal health coverage: the strange romance of the Lancet, MEDICC, and Cuba. Social Medicine. 2016;9(2):93–7.

[10] Birn AE, Nervi L, Siqueira E (2016). Neoliberalism redux: the global health policy agenda and the politics of cooptation in Latin America and beyond. Dev Chang.

[11] People’s Health Movement, Medact, Third World Network, Health Poverty Action, Medico International, & ALAMES (eds.). Global Health Watch 5: An Alternative World Health Report. London: Zed Books; 2017.

[12] McKee M, Stuckler D (2018). Revisiting the corporate and commercial determinants of health. Am J Public Health.

[13] Anaf J, Baum F, Fisher M, Friel S. Civil society action against transnational corporations: implications for health promotion. Health Promot Int. 2019 Aug;22. 10.1093/heapro/daz088 31504470

[14] Sanders D, Nandi S, Labonté R, Vance C, Van Damme W (2019). From primary health care to universal health coverage—one step forward and two steps back. Lancet.

[15] Birn AE, Pillay Y (2017). Holtz TH.

[16] Sigerist HE (1999). From Bismarck to Beveridge: developments and trends in social security legislation. J Public Health Policy.

[17] Franzoni JM (2008). Welfare regimes in Latin America: capturing constellations of markets, families, and policies. Latin American Politics and Society.

[18] CEPAL (ECLAC). Panorama Económico de América Latina 2012. http://www.formaciontecnicabolivia.org/webdocs/publicaciones/2013/PanoramasocialdeALATINA.pdf

[19] CEPAL (ECLAC). Panorama Económico de América Latina 2018. http://www.formaciontecnicabolivia.org/webdocs/publicaciones/2019/PanoramasocialdeALATINA.pdf

[20] Lima NT, Gerschman S, Edler FC, Suárez JM. Saúde e democracia: história e perspectivas do SUS: SciELO-Editora FIOCRUZ; 2005.

[21] García JC. La medicina estatal en América Latina (1880–1930), partes I and II. Revista Latinoamericana de Salud 1981; 1: 73–104 and 1982; 2: 102–17.

[22] Sirrs C (2019). Promoting health protection worldwide: the International Labour Organization and health systems financing, 1952–2012. International History Review.

[23] McCartney G, Hearty W, Arnot J, Popham F, Cumbers A, McMaster R (2019). Impact of political economy on population health: a systematic review of reviews. Am J Public Health.

[24] Bradby H, Thapar-Björkert S, Hamed S, Maina AB. Undoing the unspeakable: researching racism in Swedish healthcare using a participatory process to build dialogue. Health Research Policy and Systems. 2019;17:43. 10.1186/s12961-019-0443-0 PMC6480694 31014361

[25] Burström B, Burström K, Nilsson G, Tomson G, Whitehead M, Winblad U. Equity aspects of the primary health care choice reform in Sweden – a scoping review. Int J Equity Health. 2017;16:29. 10.1186/s12939-017-0524-z PMC5273847 28129771

[26] Birn AE, Krementsov N (2018). “Socialising” primary care? The Soviet Union, WHO and the 1978 Alma-Ata Conference. BMJ Global Health.

[27] Woods EM (1999). The politics of capitalism. Mon Rev.

[28] Brown W (2019). In the ruins of neoliberalism: the rise of antidemocratic politics in the west.

[29] Schrecker T (2016). Neoliberalism and health: the linkages and the dangers. Sociol Compass.

[30] ECLAC (1996). (CEPAL). The economic experience of the last fifteen years: Latin America and the Caribbean, 1980–1995.

[31] Prashad V (2013). The poorer nations: a possible history of the global south.

[32] Armada F, Muntaner C, Navarro V (2001). Health and social security reforms in Latin America: the convergence of the World Health Organization, the World Bank, and transnational corporations. Int J Health Serv.

[33] Martínez Franzoni J, Sánchez-Ancochea D (2018). Undoing segmentation? Latin American health care policy during the economic boom. Social Policy & Administration.

[34] Laurell AC (2010). Revisando las políticas y discursos en salud en América Latina. Medicina Social.

[35] Heredia N, Laurell AC, Feo O, Noronha J, González-Guzmán R, Torres-Tovar M (2015). The right to health: what model for Latin America?. Lancet.

[36] Waitzkin H (2003). Report of the WHO Commission on macroeconomics and health: a summary and critique. Lancet.

[37] People's Health Movement, Medact, Medico International, Third World Network, Health Action International, ALAMES & Health Poverty Action (eds.). Global Health Watch 2: An Alternative World Health Report. London: Zed Books; 2008.

[38] United Nations: Sustainable Development Goals. https://sustainabledevelopment.un.org/sdg3. Accessed 18 June 2019.

[39] Lethbridge JA. World Bank undermines right to universal healthcare. Bretton Woods Project 2017 Apr;6. https://www.brettonwoodsproject.org/2017/04/world-bank-undermines-right-universal-healthcare/. Accessed 16 June 2019.

[40] Giovanella L, Mendoza-Ruiz A, de Carvalho AA (2018). Universal health system and universal health coverage: assumptions and strategies. Ciência & Saúde Coletiva.

[41] Rosenau PV, Linder SH (2003). Two decades of research comparing for-profit and nonprofit health provider performance in the United States. Soc Sci Q.

[42] Devereaux PJ, Schünemann HJ, Ravindran N, Bhandari M, Garg AX, Choi PT, Grant BJ (2002). Comparison of mortality between private for-profit and private not-for-profit hemodialysis centers: a systematic review and meta-analysis. Jama..

[43] Pan American Health Organization. Health in the Americas 2017. https://www.paho.org/salud-en-las-americas-2017/?p=1993. Accessed 7 May 2019.

[44] Clarke A, Mamo L, Fosket J, Fishman J, Shim J (2010). Biomedicalization: Technoscience, health, and illness in the US.

[45] Iriart C, Merhy EE (2017). Inter-capitalistic disputes, biomedicalization and hegemonic medical model. Interface-Comunicação, Saúde, Educação.

[46] Katz AR (2013). Noncommunicable diseases: global health priority or market opportunity? An illustration of the World Health Organization at its worst and at its best. Int J Health Serv.

[47] Breilh J (2013). La determinación social de la salud como herramienta de transformación hacia una nueva salud pública (salud colectiva). Revista Facultad Nacional de Salud Pública.

[48] Jamison DT, Summers LH, Alleyne G, Arrow KJ, Berkley S, Binagwaho A, Bustreo F (2013). Global health 2035: a world converging within a generation. Lancet.

[49] Ottersen OP, Dasgupta J, Blouin C, Buss P, Chongsuvivatwong V, Frenk J, Fukuda-Parr S (2014). The political origins of health inequity: prospects for change. Lancet.

[50] Bhambra GK, Holmwood J (2018). Colonialism, postcolonialism and the liberal welfare state. New Political Economy.

[51] Rodriguez MI (Commissioned), Brito P, Campos F, Nervi L, Rovere M. Human Resources for Health as Protagonists of Health Systems Based on PHC. High-level Commission of the Universal Health in the 21st Century: 40 Years of Alma-Ata, PAHO/WHO. Report of the HLC (Group 5, Human Resources), Washington, DC; 2019.

[52] Prashad V (2019). Religion is the sigh of the oppressed creature: the twenty-seventh newsletter.

